# Propargylaminyl 3α-hy­droxy-11-oxo-18β-olean-12-en-29-oate

**DOI:** 10.1107/S1600536811043534

**Published:** 2011-10-29

**Authors:** Laszlo Czollner, Ulrich Jordis, Kurt Mereiter

**Affiliations:** a Institute of Applied Synthetic Chemistry, Vienna University of Technology, Getreidemarkt 9/163, A-1060 Vienna, Austria; b Institute of Chemical Technologies and Analytics, Vienna University of Technology, Getreidemarkt 9/164SC, A-1060 Vienna, Austria

## Abstract

The title compound, C_33_H_49_NO_3_, is the propargyl­amide of 18β-glycyrrhetinic acid, a penta­cyclic triterpenoid of inter­est as a therapeutic agent. The five six-membered rings of the glycyrrhetinic acid moiety show normal geometries, with four rings in chair conformations and the unsaturated ring *C* in a half-chair conformation. In the crystal, the terminal *N*-propargylcarboxamide group has remarkable structural effects on weak hydrogen-bond-like inter­actions. Particularly noteworthy are an inter­molecular O—H⋯π inter­action accepted side-on by the terminal alkyne group [O⋯C = 3.097 (2) and 3.356 (2) Å] and a short inter­molecular C—H⋯O inter­action [C⋯O = 3.115 (2) Å] donated by the alkyne C—H group. An N—H⋯O [N⋯O = 3.251 (2) Å] and a C_alkyl_—H⋯O [C⋯O = 3.254 (2) Å] interaction complement the crystal structure.

## Related literature

For general information on the therapeutic aspects of the parent compounds glycyrrhizin and 18β-glycyrrhetinic acid, see: Baran *et al.* (1974 ▶); Kitagawa (2002 ▶); Asl & Hosseinzadeh (2008 ▶). For the synthesis of derivatives of 18β-glycyrrhetinic acid with a therapeutic background, see: Su *et al.* (2004 ▶); Beseda *et al.* (2010 ▶). For the crystal structures of 18β-glycyrrhetinic acid and derivatives, see: Campsteyn *et al.* (1977 ▶); Alvarez-Larena *et al.* (2007 ▶); Beseda *et al.* (2010 ▶); Amer *et al.* (2010 ▶). For the crystal structure data of several *N*-propargylcarboxamides, see: Hashmi *et al.* (2004 ▶); Frey *et al.* (2008 ▶). For weak hydrogen bonds involving C C—H moieties, see: Desiraju & Steiner (1999 ▶).
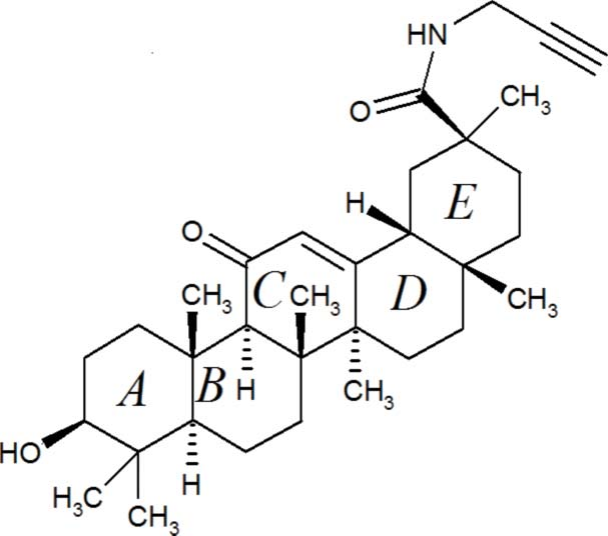


## Experimental

### 

#### Crystal data

C_33_H_49_NO_3_
*M* *_r_* = 507.73
Orthorhombic, 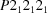

*a* = 6.7534 (8) Å
*b* = 13.4879 (16) Å
*c* = 31.132 (4) Å
*V* = 2835.8 (6) Å^3^
*Z* = 4
Mo *K*α radiation
μ = 0.07 mm^−1^
*T* = 100 K
0.56 × 0.43 × 0.38 mm

#### Data collection

Bruker Kappa APEXII CCD diffractometer
Absorption correction: multi-scan (*SADABS*; Bruker, 2008 ▶) *T*_min_ = 0.87, *T*_max_ = 0.97
41876 measured reflections
4658 independent reflections
4531 reflections with *I* > 2σ(*I*)
*R*_int_ = 0.026

#### Refinement

*R*[*F* ^2^ > 2σ(*F* ^2^)] = 0.033
*wR*(*F* ^2^) = 0.089
*S* = 1.08
4658 reflections
349 parameters
H atoms treated by a mixture of independent and constrained refinement
Δρ_max_ = 0.36 e Å^−3^
Δρ_min_ = −0.21 e Å^−3^

### 

Data collection: *APEX2* (Bruker, 2008 ▶); cell refinement: *SAINT* (Bruker, 2008 ▶); data reduction: *SAINT*, *SADABS* and *XPREP* (Bruker, 2008 ▶); program(s) used to solve structure: *SHELXS97* (Sheldrick, 2008 ▶); program(s) used to refine structure: *SHELXL97* (Sheldrick, 2008 ▶); molecular graphics: *Mercury* (Macrae *et al.*, 2006 ▶); software used to prepare material for publication: *PLATON* (Spek, 2009 ▶) and *publCIF* (Westrip, 2010 ▶).

## Supplementary Material

Crystal structure: contains datablock(s) global, I. DOI: 10.1107/S1600536811043534/jj2102sup1.cif

Structure factors: contains datablock(s) I. DOI: 10.1107/S1600536811043534/jj2102Isup2.hkl

Additional supplementary materials:  crystallographic information; 3D view; checkCIF report

## Figures and Tables

**Table 1:** Hydrogen-bond and O—H⋯π geometry (Å, °)

*D*—H⋯*A*	*D*—H	H⋯*A*	*D*⋯*A*	*D*—H⋯*A*
O1—H1*O*⋯C32^i^	0.81 (2)	2.57 (2)	3.3559 (17)	164 (2)
O1—H1*O*⋯C33^i^	0.81 (2)	2.40 (2)	3.0973 (17)	145 (2)
N1—H1*N*⋯O2^ii^	0.80 (2)	2.57 (2)	3.2511 (15)	144 (2)
C31—H31*B*⋯O1^iii^	0.99	2.56	3.2541 (17)	127
C33—H33⋯O2^iv^	0.95	2.27	3.1154 (17)	148

Symmetry codes: (i) 
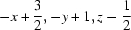
; (ii) 
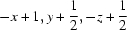
; (iii) 
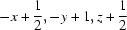
; (iv) 
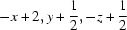
.

**Fig. 1.**
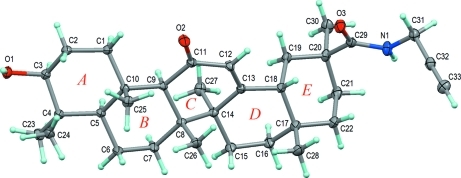
The molecular structure of (I) with displacement ellipsoids for the non-hydrogen atoms drawn at the 50% probability level. Red capitals are the ring designations.

**Fig. 2.**
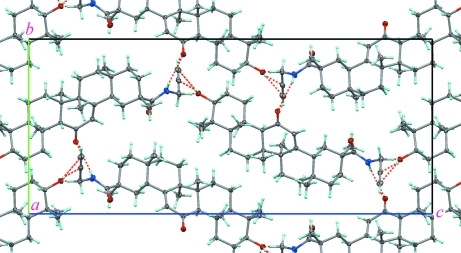
Packing diagram of (I) in a view along the a-axis. Dashed lines indicate weak O—H···C, N—H···O and C—H···O interactions outlined in section Comment.

**Fig. 3.**
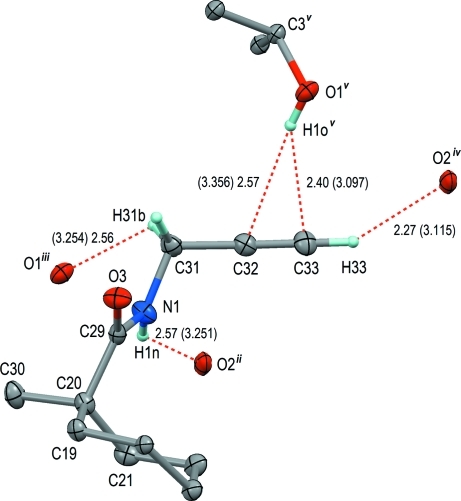
Close-up of the N-propargylcarboxamide group in (I) and its weak O—H···C, N—H···O and C—H···O interactions. Numbers with two decimal places are H···acceptor distances, numbers with three decimal places are corresponding C/N/O—acceptor distances (Å). Symmetry codes ii, iii, and iv are given in Table 1, code v is -x + 3/2, -y + 1, z + 1/2.

## supplementary crystallographic information

### Comment

18β-glycyrrhetinic acid (GA) is a pentacyclic triterpene and the aglycone of glycyrrhizin, the main sweet tasting compound from liquorice root in use as flavoring and sweetener (Kitagawa, 2002). GA is a therapeutic agent with a broad range of activity by modulating the steroid hormone cortisol (Baran *et al.*, 1974; Asl & Hosseinzadeh, 2008). One strategy to improve or modify its therapeutic profile is to leave the triterpene core of GA unaltered and to attach suitable functional groups to its 3-hydroxy group (Su *et al.*, 2004). An example for this strategy is the hydrogen succinate of GA, the licenced anti-ulcer drug Carbenoxolone. An analogous approach was used for the title compound (I), here however with the COOH group at the opposite side of GA functionalized by a propargylamide group. The synthesis of this compound and a series of relatives was recently described (Beseda *et al.*, 2010). Here we report the crystal structure of this compound. The molecular structure of (I) is shown in Fig. 1. The GA core of the molecule consists of four six-membered rings A, B, D, and E in chair conformation and the unsaturated ring C in half-chair conformation (Fig. 1). The GA core agrees well in bond lengths, bond angles, and conformation with related compounds (Campsteyn *et al.*, 1977; Alvarez-Larena *et al.*, 2007; Beseda *et al.*, 2010) and needs no further discussion. The carboxamide group O3═C29—N1 is *exo*-oriented with respect to N, C18—C20—C29—N1 = 162.3 (1)°. In case of *endo*-orientation (rare) this angle is about -30° (for examples, see: Amer *et al.*, 2010). The propargyl group has a C32≡C33 bond length of 1.197 (2) Å and an orientation defined by the torsion angle C29—N1—C31—C32 = 106.5 (1)°. In *N*-propargylcarboxamides this torsion angle varies widely (Hashmi *et al.*, 2004; Frey *et al.*, 2008). In the unit cell the molecules of (I) are aligned with their longest direction slightly inclined to the *c*-axis and adopt in this direction an undular head-to-tail-like arrangement (Fig. 2). Along the short *a*-axis (6.75 Å) the molecules are stacked directly upon each other by translation. Coherence of the structure is provided by a combination of van der Waals and weak hydrogen bond interactions listed in Table 1. Most interesting in this respect are the interactions of the *N*-propargylcarboxamide group outlined in Fig. 3. The terminal alkyne group C32≡C33—H33 has a distinctly acidic hydrogen atom and forms the by far shortest weak hydrogen bond-like interaction of the structure with distances of H33···O2^iv^ = 2.27 Å and C33···O2^iv^ = 3.115 (2) Å to the 11-keto-oxygen of the compound. After normalization (C—H = 1.083 Å; Desiraju & Steiner, 1999), the distance H33···O2^iv^ is 2.16 Å, distinctly shorter than the mean distance d(H···O) = 2.29 (3) Å reported by Desiraju & Steiner (1999; Table 2.3 of this reference) for C≡C—H···O═C< entities. The second remarkable association in Fig. 3 is the intermolecular O—H···π interaction from the hydroxy group O1^v^—H1o^v^ side-on to the two alkyne carbon atoms C32 and C33. The normalized H1o^v^···C distances (O1—H1o normalized to 0.983 Å), are 2.26 Å to C33 and 2.41 Å to C32, and correspond to the shortest O—H···π interactions reported by Desiraju & Steiner (1999; Table 3.12 of this reference). Comparable C—H···O and *X*—H···π interactions with mostly longer respective interatomic distances can be found in a small group of *N*-propargylcarboxamide containing crystal structures reported by Hashmi and coworkers in context with oxazole ring forming reactions (Hashmi *et al.*, 2004; Frey *et al.*, 2008).

### Experimental

The synthesis and properties of the title compound were described by Beseda *et al.* (2010). Platy colourless crystals for X-ray diffraction were obtained from CH_2_Cl_2_ by diethyl ether vapour diffusion at ambient temperature.

### Refinement

The two N– and O–bonded hydrogen atoms were located by a Fourier map and were the refined in *x, y, z*, and *U*_iso_. All C-bonded H atoms were placed in calculated positions and thereafter treated as riding with CH = 1.00 Å, CH_2_ = 0.99 Å and CH_3_ = 0.98 Å. A torsional parameter was refined for each methyl group. *U*_iso_(H) = 1.2*U*_eq_(C_non-methyl_) and *U*_iso_(H) = 1.5*U*_eq_(C_methyl_) were used. Because of insignificant anomalous dispersion effects, the 3501 Friedel pairs were merged prior to the final refinement. The absolute structure of the parent compound 18β-glycyrrhetinic acid is known.

### Crystal data

**Table:**

C_33_H_49_NO_3_	*F*(000) = 1112
*M**_r_* = 507.73	*D*_x_ = 1.189 Mg m^−^^3^
Orthorhombic, *P*2_1_2_1_2_1_	Mo *K*α radiation, λ = 0.71073 Å
Hall symbol: P 2ac 2ab	Cell parameters from 9975 reflections
*a* = 6.7534 (8) Å	θ = 2.5–31.0°
*b* = 13.4879 (16) Å	µ = 0.07 mm^−^^1^
*c* = 31.132 (4) Å	*T* = 100 K
*V* = 2835.8 (6) Å^3^	Block, colourless
*Z* = 4	0.56 × 0.43 × 0.38 mm

### Data collection

**Table:**

Bruker Kappa APEXII CCD diffractometer	4658 independent reflections
Radiation source: fine-focus sealed tube	4531 reflections with *I* > 2σ(*I*)
graphite	*R*_int_ = 0.026
φ and ω scans	θ_max_ = 30.0°, θ_min_ = 3.0°
Absorption correction: multi-scan (*SADABS*; Bruker, 2008)	*h* = −9→9
*T*_min_ = 0.87, *T*_max_ = 0.97	*k* = −18→18
41876 measured reflections	*l* = −43→43

### Refinement

**Table:**

Refinement on *F*^2^	Primary atom site location: structure-invariant direct methods
Least-squares matrix: full	Secondary atom site location: difference Fourier map
*R*[*F*^2^ > 2σ(*F*^2^)] = 0.033	Hydrogen site location: difference Fourier map
*wR*(*F*^2^) = 0.089	H atoms treated by a mixture of independent and constrained refinement
*S* = 1.08	*w* = 1/[σ^2^(*F*_o_^2^) + (0.0593*P*)^2^ + 0.405*P*] where *P* = (*F*_o_^2^ + 2*F*_c_^2^)/3
4658 reflections	(Δ/σ)_max_ < 0.001
349 parameters	Δρ_max_ = 0.36 e Å^−^^3^
0 restraints	Δρ_min_ = −0.21 e Å^−^^3^

### Special details

**Table:**

Geometry. All e.s.d.'s are estimated using the full covariance matrix. The cell e.s.d.'s are taken into account individually in the estimation of e.s.d.'s in distances, angles and torsion angles; correlations between e.s.d.'s in cell parameters are only used when they are defined by crystal symmetry.
Refinement. Refinement of *F*^2^ against ALL reflections. The weighted *R*-factor *wR* and goodness of fit *S* are based on *F*^2^, conventional *R*-factors *R* are based on *F*, with *F* set to zero for negative *F*^2^. The threshold expression of *F*^2^ > σ(*F*^2^) is used only for calculating *R*-factors(gt) *etc*. and is not relevant to the choice of reflections for refinement. *R*-factors based on *F*^2^ are statistically about twice as large as those based on *F*, and *R*- factors based on ALL data will be even larger.

### Fractional atomic coordinates and isotropic or equivalent isotropic displacement parameters (Å^2^)

**Table:**

	*x*	*y*	*z*	*U*_iso_*/*U*_eq_	
O1	0.33309 (16)	0.31149 (7)	−0.07972 (3)	0.02015 (19)	
H1O	0.442 (4)	0.2913 (16)	−0.0864 (7)	0.037 (6)*	
O2	0.69829 (15)	0.41262 (7)	0.11669 (3)	0.02086 (19)	
O3	0.54563 (16)	0.58041 (7)	0.29778 (3)	0.0230 (2)	
N1	0.44107 (17)	0.70328 (9)	0.34128 (3)	0.0200 (2)	
H1N	0.359 (4)	0.7444 (15)	0.3467 (6)	0.036 (5)*	
C1	0.48914 (18)	0.36216 (8)	0.03539 (3)	0.0145 (2)	
H1A	0.5902	0.3298	0.0537	0.017*	
H1B	0.3582	0.3510	0.0489	0.017*	
C2	0.49087 (19)	0.31341 (8)	−0.00911 (4)	0.0151 (2)	
H2A	0.6258	0.3179	−0.0214	0.018*	
H2B	0.4570	0.2423	−0.0062	0.018*	
C3	0.34512 (18)	0.36236 (8)	−0.03949 (4)	0.0145 (2)	
H3	0.2116	0.3569	−0.0258	0.017*	
C4	0.38603 (19)	0.47405 (8)	−0.04565 (4)	0.0146 (2)	
C5	0.38760 (18)	0.52135 (8)	0.00015 (3)	0.0134 (2)	
H5	0.2524	0.5076	0.0118	0.016*	
C6	0.4025 (2)	0.63474 (9)	−0.00030 (4)	0.0185 (2)	
H6A	0.5416	0.6548	−0.0053	0.022*	
H6B	0.3206	0.6617	−0.0239	0.022*	
C7	0.3310 (2)	0.67657 (9)	0.04257 (4)	0.0189 (2)	
H7A	0.1891	0.6601	0.0463	0.023*	
H7B	0.3428	0.7497	0.0419	0.023*	
C8	0.44738 (18)	0.63652 (8)	0.08139 (3)	0.0136 (2)	
C9	0.47071 (17)	0.52100 (8)	0.07863 (3)	0.01207 (19)	
H9	0.3343	0.4950	0.0840	0.014*	
C10	0.53109 (17)	0.47525 (8)	0.03395 (3)	0.01207 (19)	
C11	0.59034 (18)	0.48589 (8)	0.11744 (3)	0.0141 (2)	
C12	0.57055 (18)	0.54248 (8)	0.15768 (4)	0.0150 (2)	
H12	0.6418	0.5195	0.1820	0.018*	
C13	0.45862 (17)	0.62445 (8)	0.16244 (3)	0.0131 (2)	
C14	0.33519 (18)	0.66155 (8)	0.12473 (4)	0.0138 (2)	
C15	0.2914 (2)	0.77451 (9)	0.12719 (4)	0.0201 (2)	
H15A	0.4054	0.8110	0.1150	0.024*	
H15B	0.1743	0.7892	0.1091	0.024*	
C16	0.2526 (2)	0.81288 (9)	0.17273 (4)	0.0206 (2)	
H16A	0.1269	0.7843	0.1834	0.025*	
H16B	0.2370	0.8858	0.1718	0.025*	
C17	0.4198 (2)	0.78659 (9)	0.20414 (4)	0.0167 (2)	
C18	0.44227 (18)	0.67238 (8)	0.20660 (3)	0.0139 (2)	
H18	0.5696	0.6588	0.2219	0.017*	
C19	0.27685 (19)	0.62085 (9)	0.23255 (4)	0.0164 (2)	
H19A	0.1517	0.6249	0.2161	0.020*	
H19B	0.3107	0.5498	0.2357	0.020*	
C20	0.24291 (18)	0.66535 (9)	0.27734 (4)	0.0163 (2)	
C21	0.2018 (2)	0.77698 (10)	0.27197 (4)	0.0203 (2)	
H21A	0.0784	0.7862	0.2553	0.024*	
H21B	0.1822	0.8074	0.3006	0.024*	
C22	0.3723 (2)	0.82909 (9)	0.24901 (4)	0.0198 (2)	
H22A	0.4924	0.8242	0.2671	0.024*	
H22B	0.3393	0.9003	0.2460	0.024*	
C23	0.2125 (2)	0.51640 (10)	−0.07188 (4)	0.0226 (3)	
H23A	0.1912	0.4754	−0.0974	0.034*	
H23B	0.2435	0.5844	−0.0807	0.034*	
H23C	0.0923	0.5164	−0.0542	0.034*	
C24	0.5763 (2)	0.49108 (10)	−0.07175 (4)	0.0205 (2)	
H24A	0.5542	0.4710	−0.1016	0.031*	
H24B	0.6841	0.4516	−0.0595	0.031*	
H24C	0.6117	0.5615	−0.0708	0.031*	
C25	0.75367 (19)	0.49072 (9)	0.02375 (4)	0.0169 (2)	
H25A	0.8296	0.4909	0.0506	0.025*	
H25B	0.7716	0.5542	0.0090	0.025*	
H25C	0.8005	0.4368	0.0053	0.025*	
C26	0.6530 (2)	0.68636 (9)	0.08061 (4)	0.0188 (2)	
H26A	0.7187	0.6717	0.0533	0.028*	
H26B	0.7334	0.6608	0.1044	0.028*	
H26C	0.6375	0.7582	0.0837	0.028*	
C27	0.13253 (18)	0.60715 (10)	0.12792 (4)	0.0190 (2)	
H27A	0.0469	0.6422	0.1483	0.028*	
H27B	0.1538	0.5391	0.1379	0.028*	
H27C	0.0693	0.6060	0.0996	0.028*	
C28	0.6161 (2)	0.83234 (10)	0.18927 (4)	0.0232 (3)	
H28A	0.6539	0.8038	0.1615	0.035*	
H28B	0.7194	0.8182	0.2105	0.035*	
H28C	0.6005	0.9042	0.1863	0.035*	
C29	0.42524 (18)	0.64604 (9)	0.30565 (4)	0.0158 (2)	
C30	0.0673 (2)	0.61229 (11)	0.29881 (4)	0.0229 (3)	
H30A	−0.0528	0.6233	0.2818	0.034*	
H30B	0.0480	0.6388	0.3278	0.034*	
H30C	0.0948	0.5411	0.3005	0.034*	
C31	0.5958 (2)	0.68737 (9)	0.37267 (4)	0.0198 (2)	
H31A	0.6567	0.6216	0.3676	0.024*	
H31B	0.5365	0.6868	0.4017	0.024*	
C32	0.7511 (2)	0.76365 (9)	0.37105 (4)	0.0189 (2)	
C33	0.8791 (2)	0.82502 (10)	0.37026 (4)	0.0225 (2)	
H33	0.9807	0.8737	0.3696	0.027*	

### Atomic displacement parameters (Å^2^)

**Table:**

	*U*^11^	*U*^22^	*U*^33^	*U*^12^	*U*^13^	*U*^23^
O1	0.0240 (5)	0.0186 (4)	0.0179 (4)	0.0004 (4)	−0.0040 (3)	−0.0042 (3)
O2	0.0255 (5)	0.0196 (4)	0.0175 (4)	0.0108 (4)	−0.0036 (4)	−0.0009 (3)
O3	0.0276 (5)	0.0207 (4)	0.0207 (4)	0.0077 (4)	−0.0027 (4)	−0.0045 (3)
N1	0.0178 (5)	0.0238 (5)	0.0186 (4)	0.0035 (4)	−0.0015 (4)	−0.0069 (4)
C1	0.0187 (5)	0.0115 (4)	0.0132 (4)	0.0004 (4)	0.0004 (4)	0.0013 (4)
C2	0.0184 (5)	0.0123 (4)	0.0147 (5)	0.0009 (4)	−0.0007 (4)	0.0011 (4)
C3	0.0163 (5)	0.0132 (4)	0.0139 (4)	−0.0009 (4)	−0.0012 (4)	−0.0006 (4)
C4	0.0186 (5)	0.0127 (4)	0.0125 (4)	−0.0004 (4)	−0.0012 (4)	0.0009 (4)
C5	0.0166 (5)	0.0116 (4)	0.0120 (4)	0.0009 (4)	−0.0014 (4)	0.0013 (4)
C6	0.0302 (6)	0.0117 (4)	0.0137 (4)	0.0015 (5)	−0.0027 (5)	0.0021 (4)
C7	0.0284 (6)	0.0135 (5)	0.0148 (5)	0.0057 (5)	−0.0044 (5)	0.0005 (4)
C8	0.0173 (5)	0.0105 (4)	0.0131 (4)	0.0012 (4)	−0.0013 (4)	0.0010 (4)
C9	0.0130 (4)	0.0118 (4)	0.0114 (4)	0.0018 (4)	−0.0004 (4)	0.0004 (4)
C10	0.0122 (4)	0.0118 (4)	0.0122 (4)	0.0009 (4)	0.0001 (4)	0.0008 (4)
C11	0.0158 (5)	0.0135 (4)	0.0132 (4)	0.0015 (4)	−0.0008 (4)	0.0009 (4)
C12	0.0170 (5)	0.0155 (5)	0.0126 (4)	0.0037 (4)	−0.0016 (4)	0.0005 (4)
C13	0.0130 (5)	0.0128 (4)	0.0133 (4)	−0.0001 (4)	−0.0003 (4)	0.0005 (4)
C14	0.0148 (5)	0.0125 (4)	0.0142 (4)	0.0024 (4)	−0.0018 (4)	−0.0007 (4)
C15	0.0289 (6)	0.0142 (5)	0.0172 (5)	0.0071 (5)	−0.0030 (5)	−0.0007 (4)
C16	0.0280 (6)	0.0150 (5)	0.0188 (5)	0.0068 (5)	−0.0025 (5)	−0.0022 (4)
C17	0.0207 (5)	0.0133 (5)	0.0162 (5)	0.0005 (4)	−0.0008 (4)	−0.0023 (4)
C18	0.0154 (5)	0.0126 (4)	0.0135 (4)	0.0004 (4)	−0.0005 (4)	−0.0013 (4)
C19	0.0169 (5)	0.0178 (5)	0.0147 (4)	−0.0019 (4)	0.0013 (4)	−0.0036 (4)
C20	0.0148 (5)	0.0189 (5)	0.0153 (4)	−0.0005 (4)	0.0012 (4)	−0.0038 (4)
C21	0.0213 (6)	0.0209 (6)	0.0186 (5)	0.0059 (5)	0.0008 (5)	−0.0053 (4)
C22	0.0273 (6)	0.0148 (5)	0.0174 (5)	0.0007 (5)	−0.0004 (5)	−0.0036 (4)
C23	0.0303 (7)	0.0190 (5)	0.0183 (5)	0.0053 (5)	−0.0091 (5)	0.0003 (4)
C24	0.0279 (6)	0.0184 (5)	0.0152 (5)	−0.0057 (5)	0.0041 (5)	0.0009 (4)
C25	0.0135 (5)	0.0195 (5)	0.0177 (5)	−0.0019 (4)	0.0014 (4)	−0.0015 (4)
C26	0.0223 (6)	0.0161 (5)	0.0179 (5)	−0.0051 (5)	0.0020 (4)	0.0000 (4)
C27	0.0132 (5)	0.0236 (5)	0.0200 (5)	0.0022 (4)	−0.0012 (4)	−0.0044 (4)
C28	0.0292 (7)	0.0172 (5)	0.0231 (6)	−0.0062 (5)	0.0022 (5)	−0.0015 (5)
C29	0.0166 (5)	0.0157 (5)	0.0150 (5)	−0.0029 (4)	0.0024 (4)	−0.0007 (4)
C30	0.0176 (5)	0.0302 (6)	0.0210 (5)	−0.0046 (5)	0.0048 (4)	−0.0044 (5)
C31	0.0201 (6)	0.0225 (6)	0.0168 (5)	−0.0013 (5)	−0.0017 (5)	−0.0021 (4)
C32	0.0186 (5)	0.0210 (5)	0.0171 (5)	0.0027 (5)	0.0013 (4)	−0.0032 (4)
C33	0.0189 (6)	0.0220 (5)	0.0266 (6)	0.0021 (5)	0.0025 (5)	−0.0028 (5)

### Geometric parameters (Å, °)

**Table:**

O1—C3	1.4303 (14)	C16—C17	1.5354 (18)
O1—H1O	0.81 (2)	C16—H16A	0.9900
O2—C11	1.2284 (14)	C16—H16B	0.9900
O3—C29	1.2266 (15)	C17—C28	1.5339 (19)
N1—C29	1.3557 (15)	C17—C22	1.5438 (16)
N1—C31	1.4468 (17)	C17—C18	1.5498 (16)
N1—H1N	0.80 (2)	C18—C19	1.5439 (17)
C1—C2	1.5335 (15)	C18—H18	1.0000
C1—C10	1.5521 (15)	C19—C20	1.5352 (16)
C1—H1A	0.9900	C19—H19A	0.9900
C1—H1B	0.9900	C19—H19B	0.9900
C2—C3	1.5165 (16)	C20—C29	1.5366 (17)
C2—H2A	0.9900	C20—C30	1.5381 (18)
C2—H2B	0.9900	C20—C21	1.5403 (18)
C3—C4	1.5434 (15)	C21—C22	1.5269 (19)
C3—H3	1.0000	C21—H21A	0.9900
C4—C24	1.5373 (17)	C21—H21B	0.9900
C4—C23	1.5383 (18)	C22—H22A	0.9900
C4—C5	1.5622 (15)	C22—H22B	0.9900
C5—C6	1.5328 (15)	C23—H23A	0.9800
C5—C10	1.5597 (15)	C23—H23B	0.9800
C5—H5	1.0000	C23—H23C	0.9800
C6—C7	1.5276 (16)	C24—H24A	0.9800
C6—H6A	0.9900	C24—H24B	0.9800
C6—H6B	0.9900	C24—H24C	0.9800
C7—C8	1.5397 (16)	C25—H25A	0.9800
C7—H7A	0.9900	C25—H25B	0.9800
C7—H7B	0.9900	C25—H25C	0.9800
C8—C26	1.5429 (17)	C26—H26A	0.9800
C8—C9	1.5685 (15)	C26—H26B	0.9800
C8—C14	1.5837 (16)	C26—H26C	0.9800
C9—C11	1.5286 (15)	C27—H27A	0.9800
C9—C10	1.5754 (15)	C27—H27B	0.9800
C9—H9	1.0000	C27—H27C	0.9800
C10—C25	1.5505 (17)	C28—H28A	0.9800
C11—C12	1.4731 (15)	C28—H28B	0.9800
C12—C13	1.3475 (15)	C28—H28C	0.9800
C12—H12	0.9500	C30—H30A	0.9800
C13—C18	1.5232 (15)	C30—H30B	0.9800
C13—C14	1.5243 (15)	C30—H30C	0.9800
C14—C15	1.5539 (16)	C31—C32	1.4697 (18)
C14—C27	1.5561 (17)	C31—H31A	0.9900
C15—C16	1.5318 (17)	C31—H31B	0.9900
C15—H15A	0.9900	C32—C33	1.1973 (19)
C15—H15B	0.9900	C33—H33	0.9500
			
C3—O1—H1O	109.5 (15)	H16A—C16—H16B	107.8
C29—N1—C31	121.67 (11)	C28—C17—C16	110.53 (10)
C29—N1—H1N	120.6 (15)	C28—C17—C22	107.66 (10)
C31—N1—H1N	117.5 (15)	C16—C17—C22	109.74 (10)
C2—C1—C10	113.20 (9)	C28—C17—C18	109.28 (11)
C2—C1—H1A	108.9	C16—C17—C18	109.45 (10)
C10—C1—H1A	108.9	C22—C17—C18	110.16 (9)
C2—C1—H1B	108.9	C13—C18—C19	109.49 (9)
C10—C1—H1B	108.9	C13—C18—C17	112.60 (9)
H1A—C1—H1B	107.8	C19—C18—C17	113.74 (10)
C3—C2—C1	111.83 (10)	C13—C18—H18	106.9
C3—C2—H2A	109.3	C19—C18—H18	106.9
C1—C2—H2A	109.3	C17—C18—H18	106.9
C3—C2—H2B	109.3	C20—C19—C18	114.03 (9)
C1—C2—H2B	109.3	C20—C19—H19A	108.7
H2A—C2—H2B	107.9	C18—C19—H19A	108.7
O1—C3—C2	111.97 (9)	C20—C19—H19B	108.7
O1—C3—C4	111.69 (9)	C18—C19—H19B	108.7
C2—C3—C4	112.72 (10)	H19A—C19—H19B	107.6
O1—C3—H3	106.7	C19—C20—C29	109.58 (10)
C2—C3—H3	106.7	C19—C20—C30	109.15 (10)
C4—C3—H3	106.7	C29—C20—C30	106.84 (10)
C24—C4—C23	107.49 (10)	C19—C20—C21	108.10 (10)
C24—C4—C3	111.17 (10)	C29—C20—C21	111.87 (10)
C23—C4—C3	106.98 (10)	C30—C20—C21	111.27 (11)
C24—C4—C5	114.57 (10)	C22—C21—C20	111.39 (10)
C23—C4—C5	109.76 (10)	C22—C21—H21A	109.4
C3—C4—C5	106.64 (9)	C20—C21—H21A	109.4
C6—C5—C10	111.30 (10)	C22—C21—H21B	109.4
C6—C5—C4	113.55 (9)	C20—C21—H21B	109.4
C10—C5—C4	117.20 (9)	H21A—C21—H21B	108.0
C6—C5—H5	104.4	C21—C22—C17	114.17 (10)
C10—C5—H5	104.4	C21—C22—H22A	108.7
C4—C5—H5	104.4	C17—C22—H22A	108.7
C7—C6—C5	109.85 (10)	C21—C22—H22B	108.7
C7—C6—H6A	109.7	C17—C22—H22B	108.7
C5—C6—H6A	109.7	H22A—C22—H22B	107.6
C7—C6—H6B	109.7	C4—C23—H23A	109.5
C5—C6—H6B	109.7	C4—C23—H23B	109.5
H6A—C6—H6B	108.2	H23A—C23—H23B	109.5
C6—C7—C8	113.25 (10)	C4—C23—H23C	109.5
C6—C7—H7A	108.9	H23A—C23—H23C	109.5
C8—C7—H7A	108.9	H23B—C23—H23C	109.5
C6—C7—H7B	108.9	C4—C24—H24A	109.5
C8—C7—H7B	108.9	C4—C24—H24B	109.5
H7A—C7—H7B	107.7	H24A—C24—H24B	109.5
C7—C8—C26	107.12 (9)	C4—C24—H24C	109.5
C7—C8—C9	110.90 (9)	H24A—C24—H24C	109.5
C26—C8—C9	109.97 (10)	H24B—C24—H24C	109.5
C7—C8—C14	110.46 (9)	C10—C25—H25A	109.5
C26—C8—C14	110.56 (9)	C10—C25—H25B	109.5
C9—C8—C14	107.85 (9)	H25A—C25—H25B	109.5
C11—C9—C8	108.51 (9)	C10—C25—H25C	109.5
C11—C9—C10	116.09 (9)	H25A—C25—H25C	109.5
C8—C9—C10	117.61 (9)	H25B—C25—H25C	109.5
C11—C9—H9	104.3	C8—C26—H26A	109.5
C8—C9—H9	104.3	C8—C26—H26B	109.5
C10—C9—H9	104.3	H26A—C26—H26B	109.5
C25—C10—C1	108.37 (9)	C8—C26—H26C	109.5
C25—C10—C5	114.24 (9)	H26A—C26—H26C	109.5
C1—C10—C5	107.33 (9)	H26B—C26—H26C	109.5
C25—C10—C9	112.28 (9)	C14—C27—H27A	109.5
C1—C10—C9	108.19 (9)	C14—C27—H27B	109.5
C5—C10—C9	106.18 (9)	H27A—C27—H27B	109.5
O2—C11—C12	119.14 (10)	C14—C27—H27C	109.5
O2—C11—C9	123.22 (10)	H27A—C27—H27C	109.5
C12—C11—C9	117.63 (10)	H27B—C27—H27C	109.5
C13—C12—C11	124.71 (10)	C17—C28—H28A	109.5
C13—C12—H12	117.6	C17—C28—H28B	109.5
C11—C12—H12	117.6	H28A—C28—H28B	109.5
C12—C13—C18	119.21 (10)	C17—C28—H28C	109.5
C12—C13—C14	119.44 (10)	H28A—C28—H28C	109.5
C18—C13—C14	121.08 (9)	H28B—C28—H28C	109.5
C13—C14—C15	112.83 (9)	O3—C29—N1	121.48 (12)
C13—C14—C27	106.08 (9)	O3—C29—C20	122.60 (11)
C15—C14—C27	106.96 (10)	N1—C29—C20	115.86 (11)
C13—C14—C8	108.94 (9)	C20—C30—H30A	109.5
C15—C14—C8	110.00 (9)	C20—C30—H30B	109.5
C27—C14—C8	112.00 (9)	H30A—C30—H30B	109.5
C16—C15—C14	114.17 (10)	C20—C30—H30C	109.5
C16—C15—H15A	108.7	H30A—C30—H30C	109.5
C14—C15—H15A	108.7	H30B—C30—H30C	109.5
C16—C15—H15B	108.7	N1—C31—C32	112.85 (11)
C14—C15—H15B	108.7	N1—C31—H31A	109.0
H15A—C15—H15B	107.6	C32—C31—H31A	109.0
C15—C16—C17	112.68 (11)	N1—C31—H31B	109.0
C15—C16—H16A	109.1	C32—C31—H31B	109.0
C17—C16—H16A	109.1	H31A—C31—H31B	107.8
C15—C16—H16B	109.1	C33—C32—C31	178.94 (15)
C17—C16—H16B	109.1	C32—C33—H33	180.0
			
C10—C1—C2—C3	−56.26 (13)	C12—C13—C14—C27	88.45 (13)
C1—C2—C3—O1	−174.64 (9)	C18—C13—C14—C27	−85.52 (12)
C1—C2—C3—C4	58.41 (13)	C12—C13—C14—C8	−32.28 (14)
O1—C3—C4—C24	−56.59 (13)	C18—C13—C14—C8	153.74 (10)
C2—C3—C4—C24	70.50 (12)	C7—C8—C14—C13	−177.79 (9)
O1—C3—C4—C23	60.48 (13)	C26—C8—C14—C13	−59.40 (11)
C2—C3—C4—C23	−172.42 (10)	C9—C8—C14—C13	60.87 (12)
O1—C3—C4—C5	177.88 (10)	C7—C8—C14—C15	−53.63 (13)
C2—C3—C4—C5	−55.02 (12)	C26—C8—C14—C15	64.75 (12)
C24—C4—C5—C6	63.27 (14)	C9—C8—C14—C15	−174.98 (10)
C23—C4—C5—C6	−57.75 (14)	C7—C8—C14—C27	65.18 (12)
C3—C4—C5—C6	−173.29 (11)	C26—C8—C14—C27	−176.43 (9)
C24—C4—C5—C10	−68.75 (13)	C9—C8—C14—C27	−56.16 (12)
C23—C4—C5—C10	170.23 (10)	C13—C14—C15—C16	−37.49 (16)
C3—C4—C5—C10	54.69 (13)	C27—C14—C15—C16	78.78 (13)
C10—C5—C6—C7	−65.10 (14)	C8—C14—C15—C16	−159.35 (11)
C4—C5—C6—C7	160.06 (10)	C14—C15—C16—C17	54.15 (15)
C5—C6—C7—C8	58.01 (14)	C15—C16—C17—C28	60.43 (13)
C6—C7—C8—C26	73.20 (12)	C15—C16—C17—C22	179.01 (10)
C6—C7—C8—C9	−46.81 (14)	C15—C16—C17—C18	−59.98 (14)
C6—C7—C8—C14	−166.33 (10)	C12—C13—C18—C19	−85.20 (13)
C7—C8—C9—C11	178.96 (10)	C14—C13—C18—C19	88.79 (12)
C26—C8—C9—C11	60.67 (12)	C12—C13—C18—C17	147.22 (12)
C14—C8—C9—C11	−59.97 (12)	C14—C13—C18—C17	−38.79 (15)
C7—C8—C9—C10	44.66 (14)	C28—C17—C18—C13	−70.48 (12)
C26—C8—C9—C10	−73.64 (12)	C16—C17—C18—C13	50.69 (13)
C14—C8—C9—C10	165.72 (9)	C22—C17—C18—C13	171.44 (10)
C2—C1—C10—C25	−72.74 (12)	C28—C17—C18—C19	164.22 (10)
C2—C1—C10—C5	51.10 (12)	C16—C17—C18—C19	−74.61 (12)
C2—C1—C10—C9	165.29 (10)	C22—C17—C18—C19	46.14 (14)
C6—C5—C10—C25	−65.74 (13)	C13—C18—C19—C20	−178.29 (9)
C4—C5—C10—C25	67.29 (13)	C17—C18—C19—C20	−51.35 (14)
C6—C5—C10—C1	174.08 (10)	C18—C19—C20—C29	−66.74 (13)
C4—C5—C10—C1	−52.88 (12)	C18—C19—C20—C30	176.58 (10)
C6—C5—C10—C9	58.55 (12)	C18—C19—C20—C21	55.41 (13)
C4—C5—C10—C9	−168.41 (9)	C19—C20—C21—C22	−57.84 (13)
C11—C9—C10—C25	−55.10 (13)	C29—C20—C21—C22	62.90 (13)
C8—C9—C10—C25	75.83 (12)	C30—C20—C21—C22	−177.68 (10)
C11—C9—C10—C1	64.45 (12)	C20—C21—C22—C17	57.88 (14)
C8—C9—C10—C1	−164.63 (10)	C28—C17—C22—C21	−169.30 (10)
C11—C9—C10—C5	179.40 (9)	C16—C17—C22—C21	70.36 (13)
C8—C9—C10—C5	−49.68 (13)	C18—C17—C22—C21	−50.22 (14)
C8—C9—C11—O2	−149.86 (12)	C31—N1—C29—O3	−2.50 (19)
C10—C9—C11—O2	−14.78 (17)	C31—N1—C29—C20	174.64 (11)
C8—C9—C11—C12	31.32 (14)	C19—C20—C29—O3	−20.57 (16)
C10—C9—C11—C12	166.40 (10)	C30—C20—C29—O3	97.56 (14)
O2—C11—C12—C13	179.31 (12)	C21—C20—C29—O3	−140.44 (12)
C9—C11—C12—C13	−1.82 (18)	C19—C20—C29—N1	162.32 (10)
C11—C12—C13—C18	176.53 (11)	C30—C20—C29—N1	−79.55 (13)
C11—C12—C13—C14	2.44 (18)	C21—C20—C29—N1	42.45 (14)
C12—C13—C14—C15	−154.75 (11)	C29—N1—C31—C32	106.52 (14)
C18—C13—C14—C15	31.27 (15)		

### Hydrogen-bond geometry (Å, °)

**Table:**

Please add C—H···π interaction to table

**Table:**

*D*—H···*A*	*D*—H	H···*A*	*D*···*A*	*D*—H···*A*
O1—H1O···C32^i^	0.81 (2)	2.57 (2)	3.3559 (17)	164 (2)
O1—H1O···C33^i^	0.81 (2)	2.40 (2)	3.0973 (17)	145 (2)
N1—H1N···O2^ii^	0.80 (2)	2.57 (2)	3.2511 (15)	144 (2)
C31—H31B···O1^iii^	0.99	2.56	3.2541 (17)	127.
C33—H33···O2^iv^	0.95	2.27	3.1154 (17)	148.

Symmetry codes: (i) −*x*+3/2, −*y*+1, *z*−1/2; (ii) −*x*+1, *y*+1/2, −*z*+1/2; (iii) −*x*+1/2, −*y*+1, *z*+1/2; (iv) −*x*+2, *y*+1/2, −*z*+1/2.
